# Population-level physical activity surveillance in young people: are accelerometer-based measures ready for prime time?

**DOI:** 10.1186/s12966-020-00929-4

**Published:** 2020-03-18

**Authors:** Stewart G Trost

**Affiliations:** Institute of Health and Biomedical Innovation at QLD Centre for Children’s Health Research, Queensland University of Technology, Level 6, 62 Graham Street, South Brisbane, QLD 4101 Australia

With the promotion of physical activity in young people, an established global health priority [1], it is critically important for governments and public health agencies to have a clear understanding of the proportion of children and adolescents meeting guidelines for physical activity and sedentary behaviour. Historically, the assessment of physical activity in large scale population-level surveillance systems has been limited to self-report measures. However, among youth, self-report methods are subject to significant social desirability and recall bias [2, 3]. Younger children, in particular, have difficulty recalling their past behaviour accurately; and struggle to understand the concepts of physical activity frequency, intensity, duration, and type [4]. Proxy self-reports completed by parents or caregivers are one solution, but this method is also subject to recall bias since respondents can only report on the time they are in contact with the child [2, 3]. In light of the limitations of self-report methods, device based physical activity measures such as accelerometers have become the preferred method in studies involving children and young people [2, 5].

Despite the ubiquitous use of accelerometer-based motion sensors in physical activity studies involving children and adolescents, the application of accelerometers in population-level physical activity surveillance systems has been seriously questioned [6]. Citing methodological limitations related to questionable validity, between-monitor variability, multiple sets of conflicting intensity-based cut-points, and bias resulting from monitoring non-compliance, Pedisic and Bauman [6] concluded that without appropriate standardisation protocols, the adoption of accelerometers for large-scale physical activity surveillance was premature. They further concluded that accelerometer-based physical activity measures should not substitute, but only supplement self-report information systems; and that self-report methods should be the primary assessment tool in physical activity surveillance systems.

The newly published paper by Steene-Johannessen et al. [7] goes a long way to dispel Pedisic and Bauman’s notion that accelerometer-based measures are not yet ready for “prime time” when it comes to population-level physical activity surveillance. Based on the reanalysis of accelerometer data from more than 47,000 young people from 30 different studies across 18 European countries, the authors present what could be argued as the most detailed and comprehensive descriptions of physical activity and sedentary behaviour levels in European children and adolescents to date. The results indicate that approximately two-thirds of European children and adolescents do not meet the daily 60 min moderate-to-vigorous physical activity (MVPA) recommendation, with youth from southern European countries being at significantly greater risk for inactivity than those residing in central or northern Europe. The findings are concerning to say the least and underscore the need for effective policies and programs to promote physical activity and reduce sedentary behaviour in children and young people.

Through the implementation of a carefully conceived accelerometer data processing protocol, Steene-Johannessen and colleagues [7] mitigate many of the concerns identified in the Pedisic and Bauman review [6]. The analysis was delimited to studies using an ActiGraph accelerometer on the hip, epoch length was standardised to 60 s, data from midnight to 6:00 am was excluded to minimise any bias associated with between-study variation in monitoring protocols and minimal wear time criteria, and point estimates of MVPA and sedentary behaviour were based on a single set of cut-point values, regardless of child age. Nevertheless, the numerous study limitations described in the discussion section indicate that not all the methodological limitations identified in the Pedisic and Bauman review were addressed adequately.

A significant limitation of the Steene-Johannessen study is the reliance on proprietary count-based metrics - making it impossible to integrate data from studies deploying different brands of accelerometers. The solution to this problem is to discontinue the practice of calculating and reporting count-based metrics and applying the currently available methods and metrics based on the raw acceleration signal [8–10]. It is acknowledged that Steene-Johannessen included data from studies that were conducted long before raw accelerometer data from the ActiGraph could be readily accessed, limiting their ability to use this approach. However, if future accelerometer data pooling projects are to provide legitimate between-study and cross-country comparability, the more than two decade old practice of applying cut-points to processed activity counts must be phased out.

In the Pedisic and Bauman review [6], the existence of multiple sets of conflicting intensity-based cut-points was identified as a major methodological weakness limiting the application of accelerometers in population-level surveillance studies. Steene-Johannessen and colleagues partially address this issue by applying, in a standardised manner, an intensity-based cut-point with established evidence of validity in school-aged youth [11, 12]. However, there is growing recognition that the relationship between accelerometer counts and energy expenditure is highly dependent on the activities included the calibration study; and that cut-points derived from a single regression model or Receiver Operating Characteristic curve cannot adequately characterise physical activity intensity across a wide range of physical activities [13]. In an independent evaluation of ActiGraph cut-points for youth, the Evenson thresholds were found to have the least physical activity intensity classification error of all the cut-points tested [12]. However, it is important to note that the Evenson cut-points still misclassified MVPA as light-intensity physical activity 20% of the time, and that light intensity physical activities were misclassified as sedentary at least 40% of the time [12]. Moreover, given that the relationship between activity counts and energy expenditure in children under five differs substantially to that observed in adolescent youth [14], the application of the Evenson cut-points in children aged 2- to 5-years by Steene-Johannessen must be questioned.

Over the last decade, there has been a shift from count-based thresholds to machine learning activity classification and energy expenditure estimation algorithms based on features extracted from raw accelerometer signals [15]. When applied to youth, machine learning approaches have shown to provide more accurate predictions of physical activity intensity [13, 16]. Moreover, in contrast to cut-point methods, which only estimate time spend in MVPA, physical activity classification models can predict time spent in specific activity types (e.g., walking, running, dancing, cycling) or broader activity classes (e.g., active games or sports) [13, 16]. This enables researchers in the public health and exercise sciences to explore a greater variety of physical activity metrics as well as examine age-related differences in movement behaviours that are not confounded by developmental differences in the relationship between accelerometer counts and energy expenditure.

To date, the uptake of machine learning methods by public health researchers has been slow, primarily because of the need to collect and process large quantities of raw accelerometer signal using specialised software; and partly because of concerns that machine learning models trained on laboratory-based activities trials do not generalise well to free living scenarios [17]. As machine learning accelerometer data processing methods evolve and the required computer platforms enabling public health researchers to apply machine learning methods become available, it is anticipated that future physical activity surveillance studies will address the aforementioned limitations of cut-point methods by implementing potentially more accurate and versatile machine learning accelerometer data processing methods.

In their review, Pedisic and Bauman [6] identify between-study variations in monitoring protocols and non-compliance (non-wear) as key methodological issues limiting the use of accelerometer in physical activity surveillance studies. In their study, Steene-Johannessen and colleagues [7] partially address this limitation by excluding all accelerometer data recorded from midnight to 6:00 am and excluding monitoring days with less than eight hours of valid wear time. While these decision rules are not without precedent, it is likely that at a significant percentage of participants were awake and/or wearing the monitor during the excluded time periods. Furthermore, as noted by the authors, the inclusion of studies implementing 24 h monitoring protocols may have led to an overestimation of sedentary time, given the longer daily wear periods and the misclassification of sleep as sedentary time. It can be argued that the issue of non-wear is a legacy issue from objective monitoring studies requiring participants to wear an accelerometer on the hip during the waking hours. Because hip mounted accelerometers are typically placed on snug fitting elastic belts and worn over clothing, non-compliance and insufficient wear time were frequent problems in these studies. To improve compliance and minimise missing data due to non-wear, more and more studies are implementing continuous 24-h monitoring protocols with wrist mounted accelerometers. Wrist mounted accelerometers are easier to wear for extended periods and allow investigators to evaluate compliance with more contemporary 24-h movement guidelines which require concurrent monitoring of physical activity, sedentary behaviour, and sleep [18]. With wrist mounted accelerometers, it is critically important that researchers compute physical activity metrics using more sophisticated accelerometer data processing methods [16, 19]. Simple cut-point approaches applied to either processed activity counts or raw acceleration signal (i.e., Euclidean Norm Minus One - ENMO) provide misleading estimates of movement behaviour because they do not account for upper limb movements during sedentary or stationary light-intensity activities [20, 21]. In investigations where sitting time is of primary interest, assessments of posture with thigh mounted accelerometers, alone or in combination with other placements, should be considered [22, 23].

The findings of the Steene-Johannessen study highlight the long-standing methodological issue of how to operationalise compliance with physical activity guidelines in accelerometer-based studies [24]. Steene-Johannessen and colleagues [7] operationalise meeting guidelines as accumulating an average of > 60 min of MVPA across all valid monitoring days; but other studies have applied more stringent criteria by requiring participants to accumulate ≥60 min on every monitoring day [25]. It can be argued that, based on the wording of the guidelines, children are required to reach the target of 60 min of MVPA on each and every day. If this is the case, then significantly more than two-thirds of European children and adolescents are insufficiently active for health benefit. Alternatives to the “average over all days” and “all days” methods are “the most days” methods (e.g., meeting the 60 min recommendation on > 50% of monitoring days) and the “Child x Day” method used by Cooper at colleagues [25] in which the percentage of valid monitoring days with ≥60 min of MVPA is calculated and interpreted as the probability that a randomly selected child on a randomly selected day met the guideline [24]. It is acknowledged that all methods have advantages and disadvantages. However, the difficulty associated with comparing prevalence estimates from studies applying different approaches highlights the need for consensus on how physical activity guidelines for young people should be operationised.

The article by Steene-Johannessen and colleagues [7] highlights the utility of accelerometer-based measures in physical activity surveillance studies involving children and adolescents. The findings are significant and identify the promotion of physical activity as a priority concern for public health authorities across Europe. Yet, it is important to acknowledge that the accelerometer data processing methods applied in this study have been implemented with only minor modifications for more than two decades, despite significant advances in wearable sensor technology and artificial intelligence over the same time period. If accelerometer-based measures of physical activity and sedentary behaviour are to be accepted as best practice methodology in large scale population-level surveillance studies, public health researchers must be willing to adopt more contemporary monitoring protocols and apply new accelerometer data processing methods.

## Data Availability

Not applicable
